# Construct a circRNA/miRNA/mRNA regulatory network to explore potential pathogenesis and therapy options of clear cell renal cell carcinoma

**DOI:** 10.1038/s41598-020-70484-2

**Published:** 2020-08-12

**Authors:** Shuheng Bai, YinYing Wu, Yanli Yan, Shuai Shao, Jiangzhou Zhang, Jiaxin Liu, Beina Hui, Rui Liu, Hailin Ma, Xiaozhi Zhang, Juan Ren

**Affiliations:** 1 Department of Radiotherapy, Oncology Department, First Affiliated Hospital of Xi’an Jiaotong University, Xi’an, 710061 Shaanxi Province China; 2 Medical School, Xi’an Jiaotong University, Xi’an, 710061 Shaanxi Province China; 3 Department of Chemotherapy, Oncology Department, First Affiliated Hospital of Xi’an Jiaotong University, Xi’an, 710061 Shaanxi Province China

**Keywords:** Cancer genetics, Cancer genomics, Tumour biomarkers, Computational biology and bioinformatics, Drug discovery

## Abstract

Clear cell renal cell carcinoma (ccRCC) is the most representative subtype of renal cancer. CircRNA acts as a kind of ceRNA to play a role in regulating microRNA (miRNA) in many cancers. However, the potential pathogenesis role of the regulatory network among circRNA/miRNA/mRNA is not clear and has not been fully explored. CircRNA expression profile data were obtained from GEO datasets, and the differentially expressed circRNAs (DECs) were identified through utilizing R package (Limma) firstly. Secondly, miRNAs that were regulated by these circRNAs were predicted by using Cancer-specific circRNA database and Circular RNA Interactome. Thirdly, some related genes were identified by intersecting targeted genes, which was predicted by a web tool (miRWalk) and differentially expressed genes, which was obtained from TCGA datasets. Function enrichment was analyzed, and a PPI network was constructed by Cytoscape software and DAVID web set. Subsequently, ten hub-genes were screened from the network, and the overall survival time in patients of ccRCC with abnormal expression of these hub-genes were completed by GEPIA web set. In the last, a circRNA/miRNA/mRNA regulatory network was constructed, and potential compounds and drug which may have the function of anti ccRCC were forecasted by taking advantage of CMap and PharmGKB datasets. Six DECs (hsa_circ_0029340, hsa_circ_0039238, hsa_circ_0031594, hsa_circ_0084927, hsa_circ_0035442, hsa_circ_0025135) were obtained and six miRNAs (miR-1205, miR-657, miR-587, miR-637, miR-1278, miR-548p) which are regulated by three circRNAs (hsa_circ_0084927, hsa_circ_0035442, hsa_circ_0025135) were also predicted. Then 497 overlapped genes regulated by these six miRNAs above had been predicted, and function enrichment analysis revealed these genes are mainly linked with some regulation functions of cancers. Ten hub-genes (PTGER3, ADCY2, APLN, CXCL5, GRM4, MCHR1, NPY5R, CXCR4, ACKR3, MTNR1B) have been screened from a PPI network. PTGER3, ADCY2, CXCL5, GRM4 and APLN were identified to have a significant effect on the overall survival time of patients with ccRCC. Furthermore, one compound (josamycin) and four kinds of drugs (capecitabine, hmg-coa reductase inhibitors, ace Inhibitors and bevacizumab) were confirmed as potential therapeutic options for ccRCC by CMap analysis and pharmacogenomics analysis. This study implies the potential pathogenesis of the regulatory network among circRNA/miRNA/mRNA and provides some potential therapeutic options for ccRCC.

## Introduction

Renal cancer is common cancer, and the incidence rates in males and females are 5% and 3%, respectively^1^. Clear cell renal cell carcinoma (ccRCC) accounts for 70–80% of renal cancer, which is the most representative subtype, and the incidence rate increased year by year. Compared with other cancers, kidney cancer-related clinical symptoms and biomarkers are less, so early diagnosis is difficult. Moreover, ccRCC has poor responses to conventional chemotherapy and radiation therapy, leading to a low 5-year survival rate of advanced patients, which is only 10–20%^2,3^. Nowadays, VEGF tyrosine kinase inhibitor monotherapy had been one type of standard therapy. Moreover, with the advances in immunotherapy and the more newly discovered therapeutic target, a combination of the immunotherapy and the targeted therapies could be the next standard of treatment^4^. Therefore, it is especially necessary to explore the internal mechanism of ccRCC to find some new therapeutic targets.

Circular RNA (circRNA), derived from the exon or intron region of a gene, is a particular type of non-coding RNA molecule that is different from linear RNA. Compared with linear RNA structure, circRNA has no 5′–3′ polarity and no polyA tail, making it a closed circular structure. Therefore, it is more stable than linear RNA, and it is not easily degraded by RNA exonuclease or RNase^5^. CircRNAs’ function can generalize as below (1) miRNA can regulate the post-transcriptional expression of target genes, and circRNA can act as a competing endogenous RNA (ceRNA) to bind to miRNA like a sponge to regulate the function of miRNA, thus indirectly regulating the expression of genes (2) It can affect gene expression through interacting with RNA binding protein and modulating the stability of mRNAs (3) It also can function as protein scaffolds and encode functional proteins in some cancer cells lines^6–9^. Recently, some studies have demonstrated that circRNA not only acts as a molecular marker but can also participate in cancer proliferation and invasion by regulating miRNA in colorectal cancer, lung cancer, and bladder cancer^10^.

**Figure 1:**
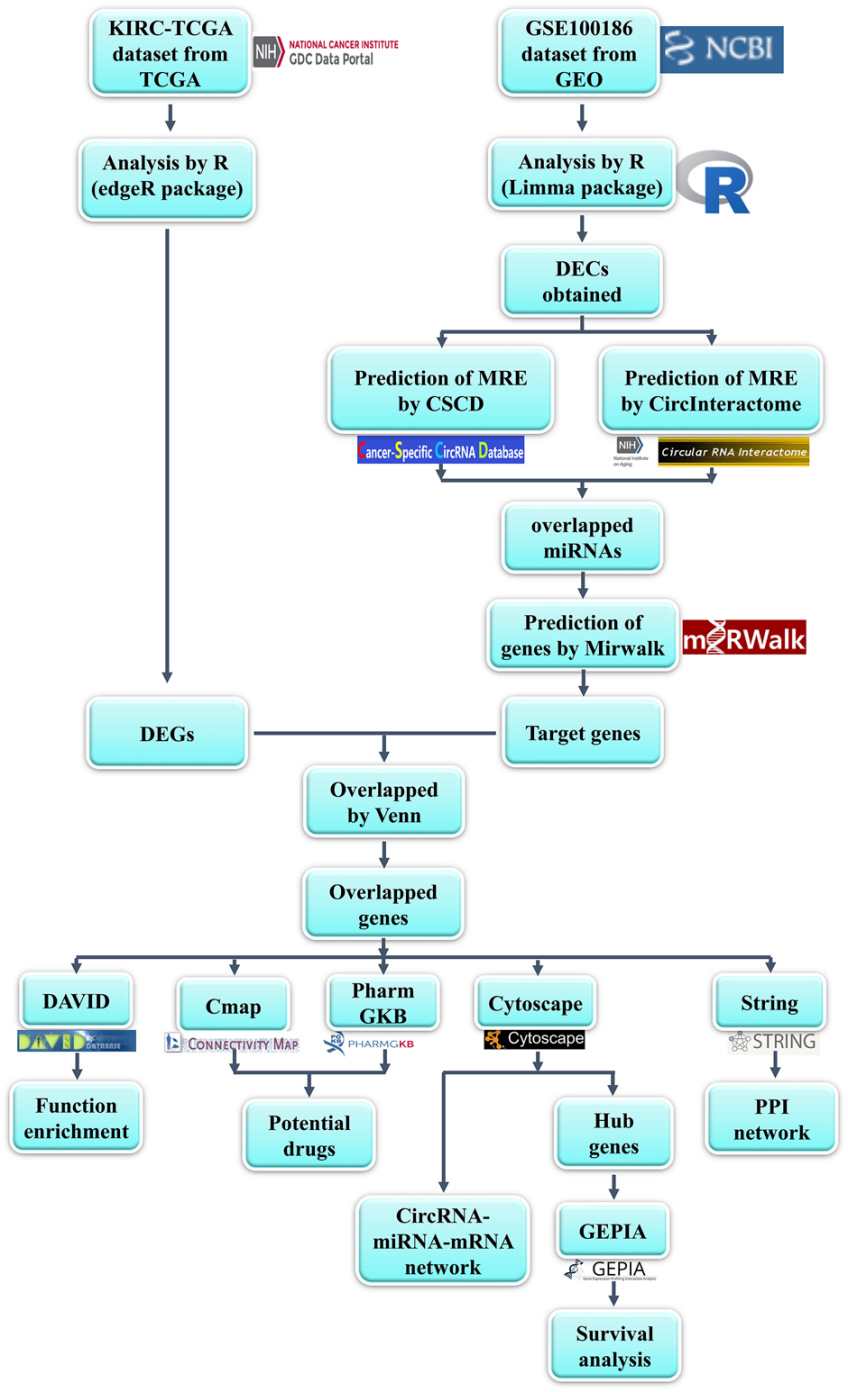
Flowchart of this study about constructing a circRNA/miRNA/mRNA network and predicting some potential therapeutic options of ccRCC.

In this study, some novel circRNAs may act as ceRNA to regulate gene expression in ccRCC, and their potential mechanisms have been investigated by utilizing gene chip and bioinformatics methods. The process digraph is showed in Fig. 1: Firstly, circRNAs related microarray datasets of ccRCC were obtained from GEO database, and differential expressed circRNAs (DECs) were also acquired. Then, to demonstrate whether the DECs function as ceRNAs in ccRCC, their related miRNAs and miRNA target genes have been collected, and a circRNA/miRNA/mRNA network also has been constructed. Furthermore, a protein–protein interaction (PPI) network was successfully built, and the hub-genes were also obtained. Functional enrichment and pathway enrichment analyses were performed to reveal the potential pathogenesis of ccRCC. Furthermore, connectivity map (CMap) analysis and pharmacogenomics analysis were conducted to predict bio-active compounds and potential drugs for the treatment of ccRCC, which may provide a new method in the latent therapeutic capacity of circRNAs in ccRCC.

## Methods

### Data obtained and DECs acquired

Gene Expression Synthesis (GEO, https://www.ncbi.nlm.nih.gov/geo/) is a public functional genomic database that allows users to query, locate, review and download research, and gene expression profiles^11^. Microarray datasets that provide circRNA expression profile data in clear cell renal cell carcinoma (ccRCC) were acquired from the GEO database. All raw expression data were normalized and log2-transformed. Then Limma, a Bioconductor package in R, was applied for differential analysis of microarray data, to determine DECs in microarray dataset with the criteria of |log2 (fold change)|> 2 and *P* value < 0.05^12^.

### Prediction of MREs

Cancer-specific circRNA database (CSCD, https://gb.whu.edu.cn/CSCD) was constructed to understand the functional effects of circRNAs, through predicting the miRNA response element (MRE) sites and RNA binding protein (RBP) sites for each circRNA^13^. Circular RNA Interactome (CircInteractome) is also a web tool to map RNA-binding proteins (RBP) and miRNA response element (MRE) sites on human circRNAs by searching some public databases of circRNA, miRNA, and RBP. It also provides bioinformatic analyses of binding sites on circRNAs, and additionally analysis of miRNA and RBP sites^14^. DIANA-miRPath v3.0 (https://www.microrna.gr/miRPathv3) is an online software that is committed to assessing miRNAs regulatory roles and forecasting the related regulation pathways^15^. The miRNA response elements (MREs), of those selected DECs, were predicted with these two web tools, CSCD and CircInteractome. Overlapped miRNAs of the two algorithms were predicted as potential target miRNAs of the DECs. DIANA-miRPath also predicted these overlapped miRNA's functions. Then these overlapped miRNAs were selected for further mRNA predictions.

### Forecasting of miRNA targeted genes

MiRWalk 2.0 is a web tool to predict miRNA–mRNA interactions. It involves 12 predicted algorithms (miRWalk, Microt4, mirbridge Targetscan, RNAhybrid, RNA22, PITA, Pictar2, miRNAMap, miRDB, miRanda and miRMap) to ensure the correctness of forecast results^16^. Then targeted genes forecasted by at least seven algorithms were selected to overlapped with differentially expressed genes (DEGs) in ccRCC from TCGA database.

### Collecting DEGs of ccRCC and obtaining the overlapped genes

The Cancer Genome Atlas (TCGA) is a public database that demonstrated major cancer related genomic alterations. Differentially expressed genes (DEGs) were determined by the edgeR package in Bioconductor with the screening criteria of |log2 (fold change)|> 2 and FDR < 0.05^17^.Then the overlapped genes between the predicted miRNA target genes and the DEGs were obtained through the Venn diagram.

### Functional enrichment analysis of overlapped genes

The database for annotation, visualization, and integrated discovery (DAVID V6.8, https://david.abcc.ncifcrf.gov/) is a freely accessed web-based online bioinformatics resource that provides tools for the functional interpretation of large lists of genes/proteins^18^. It was used to perform Gene Ontology (GO) analysis and Kyoto Encyclopedia of Genes and Genomes (KEGG) pathway enrichment analysis about the overlapped genes with a setting *P* < 0.05 and counts > 2.

### Establishment of PPI network and identification of hub-genes

The Search Tool for the Retrieval of Interacting Genes database (STRING) provides credible information on interactions between proteins and supplies detailed annotation^19^. In this study, the interactions between proteins, which has a combined score > 0.7, was considered as a statistically significant interaction. Then, a PPI network of the overlapped genes was constructed by the STRING (version 11). Cytoscape is a general-purpose, open-source software environment for the broad integration of molecular interaction network data. In molecular and biology fields, it can load molecular and genetic interaction data, integrate global datasets and functional annotations, establish powerful visual mappings across these data. A wide variety of Cytoscape Apps can enhance their capabilities^20^. Molecular Complex Detection (MCODE) is an app of Cytoscape to find densely connected regions of a vast molecular interaction network (as PPI, protein–protein-interaction network) based on node-weighting arithmetic, effectively. So, PPI networks were drawn using Cytoscape (version 3.7.1) and essential modules consisting of hub-genes and several relatively essential modules in the PPI networks identified by MCODE with selected criteria as follows: degree cut-off = 2, node score cut-off = 0.2, Max depth = 100, and k-score = 2.

### Overall survival (OS) analysis of the hub genes

The Gene Expression Profiling Interactive Analysis (GEPIA) is a web-based tool to deliver fast and customize-able functionalities based on TCGA and GTEx data. It provides key interactive and customizable functions, including differential expression analysis, profiling plotting, correlation analysis, patient survival analysis^21^. In this study, overall survival (OS) analysis was used to explore the influence on OS by differentially expressed hub-genes between ccRCC tissues and normal ones.

### Construction of circRNA–miRNA–mRNA network

A circRNA–miRNA–mRNA regulatory network was constructed by using Cytoscape software to draw the regulatory network.

### Connectivity Map (CMap) analysis

The Connectivity Map (CMap) is a gene expression profiling database and is great potential for discovering new therapeutic drugs for the disease. It is a database of biological applications in which gene expression is linked to disease, helping researchers quickly use data from gene expression profiles to compare small molecule compounds or drugs that are highly associated with disease^22^. With the help of CMap, candidate compounds would be discovered to treat the ccRCC. Negatively related drugs (*P* < 0.005 and connectivity score < 0) for anti-ccRCC were screened. These compounds' structures and some annotations were obtained from the website PubChem (https://pubchem.ncbi.nlm.nih.gov), which is an essential chemical information resource, containing 247.3 million substance descriptions, 96.5 million unique chemical structures and 237 million bioactivity test results from 1.25 million biological assays^23^.

## Pharmacogenomics analysis for hub genes

The Pharmacogenomics and Pharmacogenetics Knowledge Base (PharmGKB, https://www.pharmgkb.org/) is committed to Collecting and classifying information about how genetic variation affects drug response^24^. It is a comprehensive resource for pharmacogenetics, including their variations, pharmacodynamic pathways and their effects on drug-related phenotypes^25^. Furthermore, researchers can freely get this knowledge about potential worked drugs from this web. In this research, the network database was used to predict drugs that might act on hub-genes.

## Results

### Acquiring 6 DECs in clear cell renal cell carcinoma (ccRCC)

**Figure 2:**
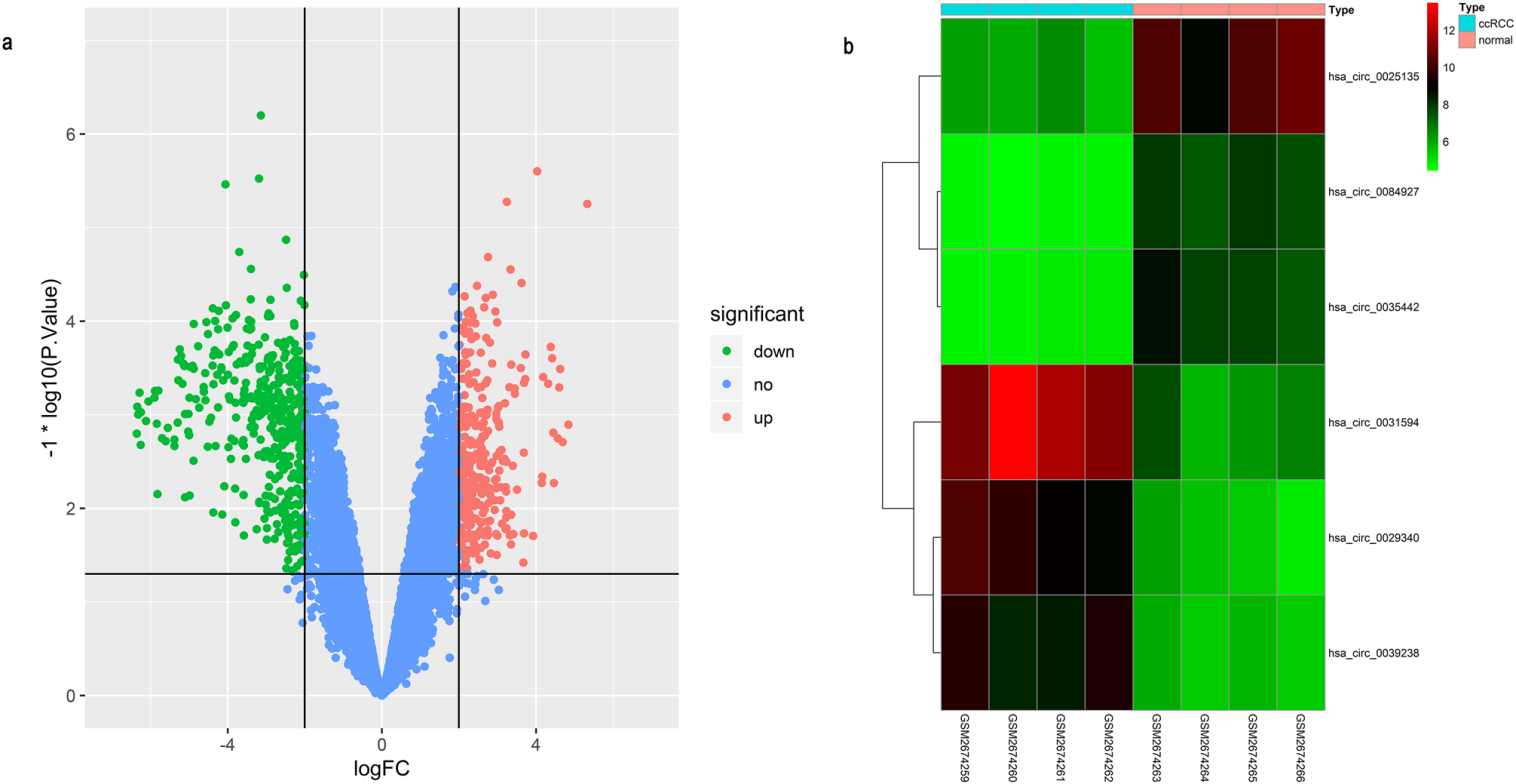
Acquire DECs of ccRCC. (**a**) Volcano plots for DECs, the green points and red points represent up and down expressed circRNAs respectively. (**b**) A heatmap for 6 differentially expressed circRNAs we selected, the change in color represents the difference in expression.

In order to explore the potential function of circRNAs and construct the interaction network between circRNAs and miRNAs in ccRCC, DECs were confirmed at the first step. A microarray dataset GSE100186 was obtained from GEO database, which includes four clear cell renal cell carcinoma tissues and four matched non-tumor tissues. The gene chip was from the platform of Agilent-074301 Arraystar Human CircRNA microarray V2. By applying Limma package, a string of circRNAs was considered as significant difference points, and six circRNAs, which shows the most credible in different expression (*P* < 0.0001 and LogFC > 2), have been selected as research objects in this study as the volcano (Fig. 2a) showing. These six circRNAs’ different expressions between 4 clear cell renal cell carcinoma tissues and 4 matched non-tumor tissues present as Fig. 2b. The basic characteristics of the 6 circRNAswere displayed in Table 1.

**Table 1:** Basic characteristics of these 6 circRNAs.

CircRNA ID	Alias	CircRNA type	Position	Strand	Best transcript	Gene symbol	Regulation
hsa_circ_0039238	hsa_circRNA_039238	exonic	Chr16:47162235–47165936	−	NM_018092	NETO2	Up
hsa_circ_0031594	hsa_circRNA_101341	exonic	chr14:34398281–34400421	−	NM_022073	EGLN3	Up
hsa_circ_0029340	hsa_circRNA_101202	exonic	chr12:125292306–125294835	−	NM_005505	SCARB1	Up
hsa_circ_0084927	hsa_circRNA_104651	exonic	chr8:95676924–95677424	+	NM_017697	ESRP1	Down
hsa_circ_0035442	hsa_circRNA_101529	exonic	chr15:58284902–58287337	−	NM_001206897	ALDH1A2	Down
hsa_circ_0025135	hsa_circRNA_101001	exonic	chr12:6458115–6465046	−	NM_001159576	SCNN1A	Down

### Identification of 6 circRNA–miRNA interactions

**Figure 3:**
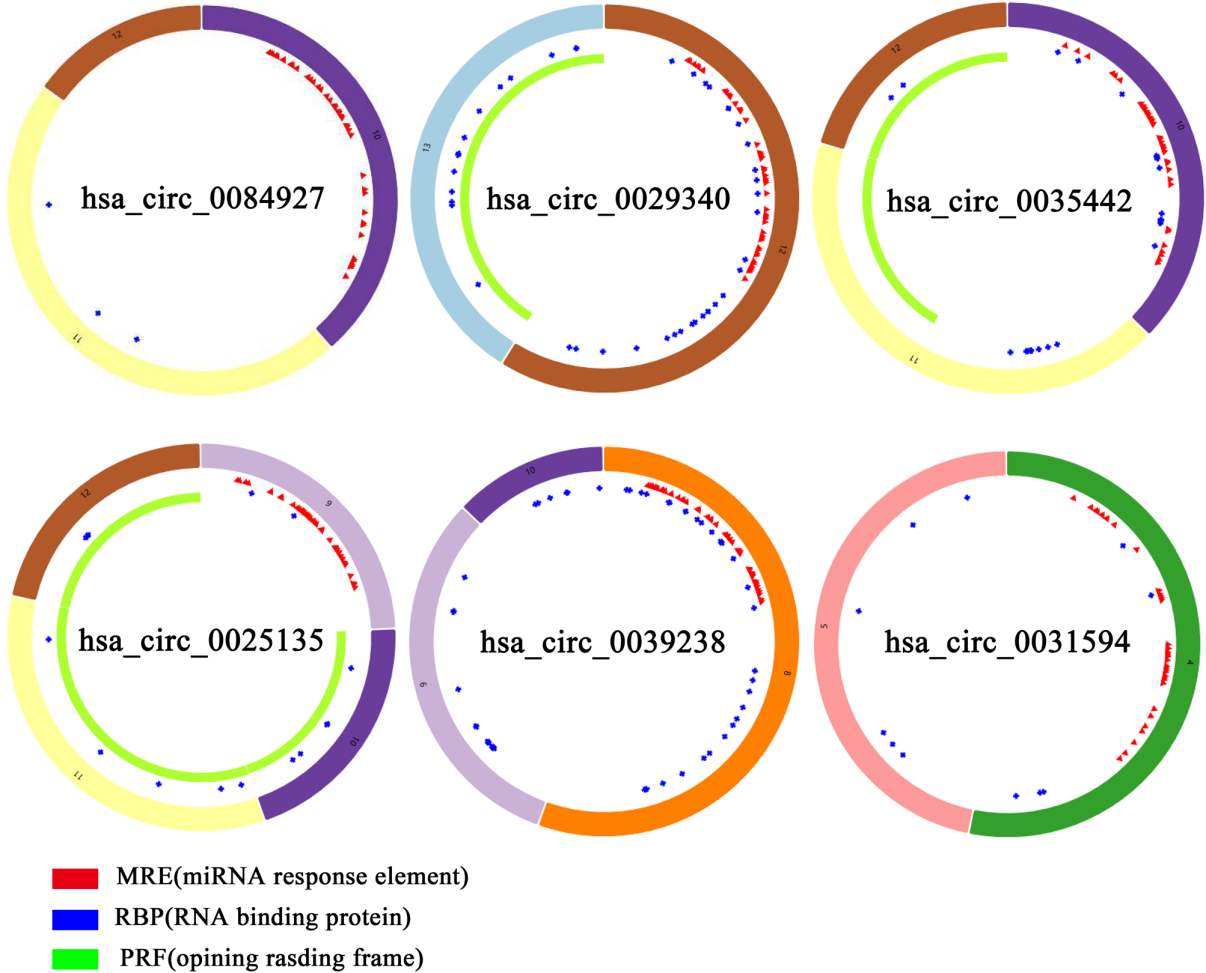
Basic structures of the 6 circRNAs. The different colors in the outer and inner ring represent the different exons and the positions of MRE, RBP and ORF.

**Figure 4:**
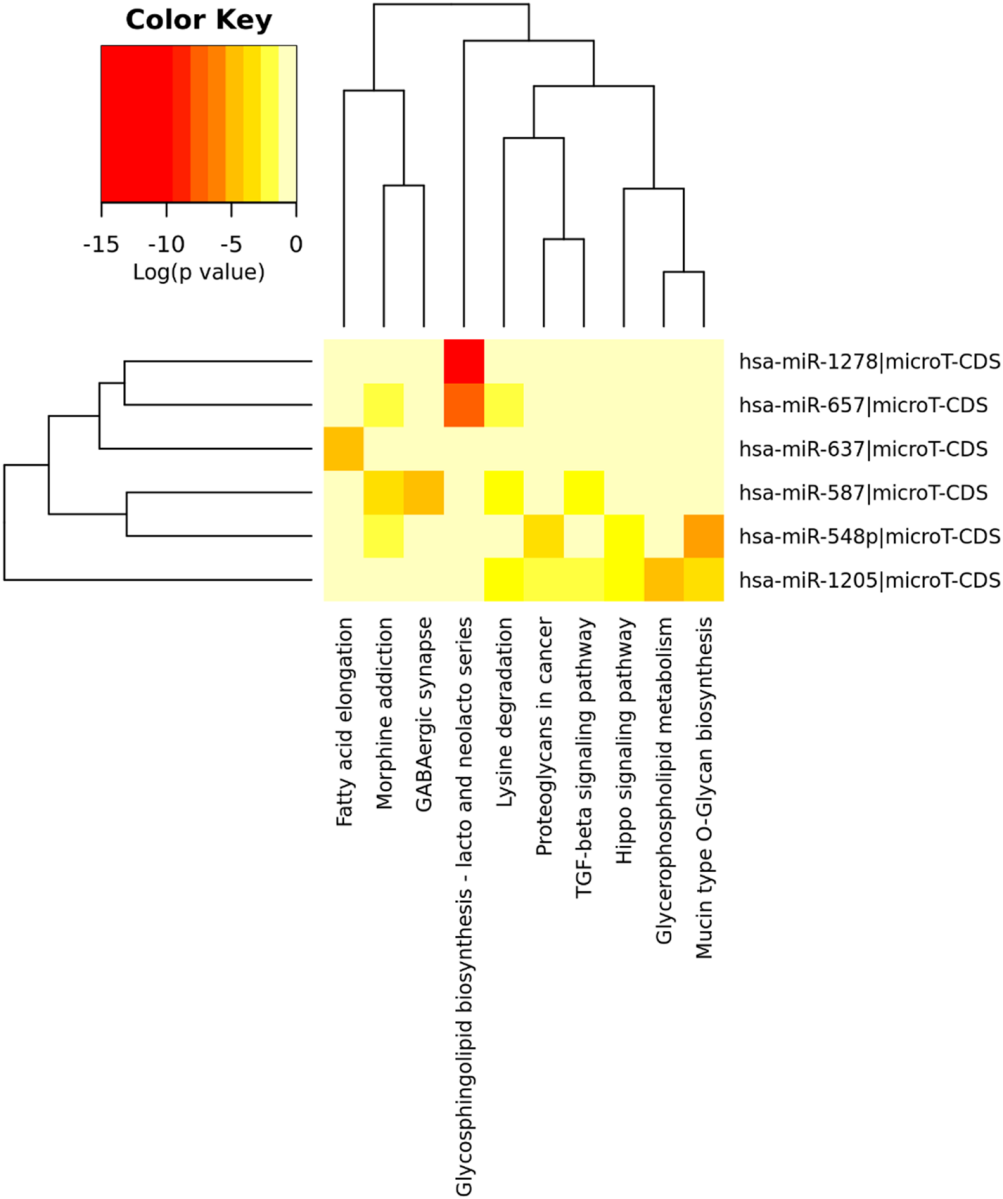
Significant signaling pathways about the 6 miRNAs by utilizing the DIANA-miRPath.

Increasing evidence has demonstrated that some circRNAs play critical roles in tumors by functioning as “sponge” to trap miRNAs. So, some miRNAs, which are related to these six DECs we got, were predicted based on this ceRNA theory. The basic structural patterns of the 6 circRNAs were illustrated in Fig. 3 which all have these structures of MRE, RBP and ORF. To explore whether all these 6 circRNAs had the function as ceRNA in ccRCC, two online databases, CSCD and CircInteractome, were used to collect and explore potential target miRNAs for them. A total of 6 circRNA–miRNA interactions, including hsa_circ_0029340-miR-1205/miR-657, hsa_circ_0025135-miR-587/miR-637 and hsa_circ_0039238-miR-1278/miR-548p, were identified. DIANA-miRPath was then used to probe the signaling pathways in that the 6 miRNAs may be involved. As shown in Fig. 4, these RNAs are involved in some pathways in the development of tumorigenesis.

### Obtaining the overlapped genes

**Figure 5:**
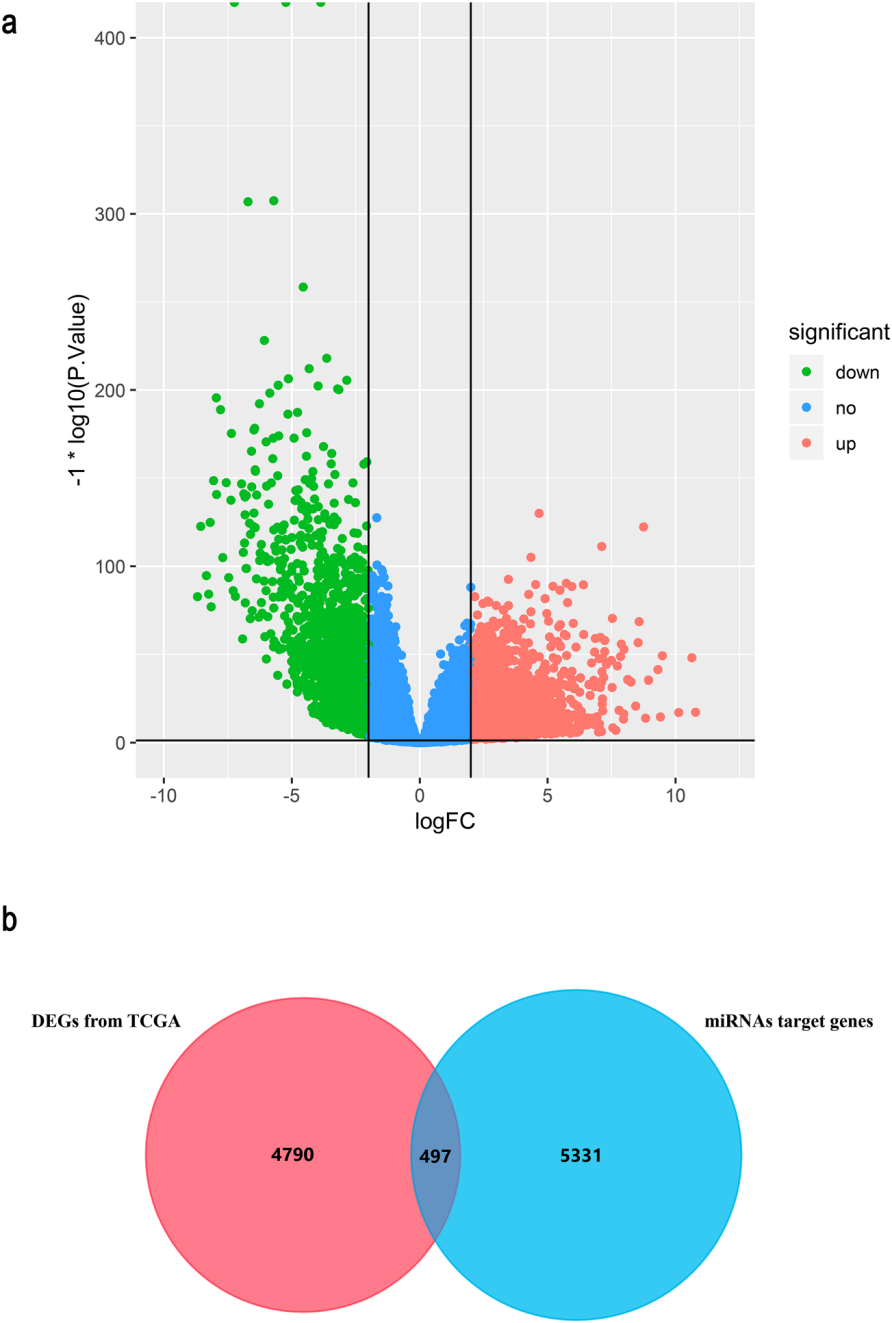
(**a**) Volcano plots showing DEGs obtained from TACG dataset by utilizing edgeR package. (**b**) Venn graph showing the 497 overlapped genes by intersecting the miRNA targeted genes and DEGs from TCGA.

Six miRNAs linked with circRNAs had been obtained. To explore these miRNAs' function in ccRCC, overlapped genes will be obtained in this work. RNA-sequencing (RNA-seq) data contained 349 clear cell renal cell carcinoma tissue samples and 49 normal controls that were obtained from the TCGA. Total 5828 DEGs in clear cell renal cell carcinoma showing in the Volcano (Fig. 5a), which were gained by running edgeR package. Moreover, 5287 target genes of the six miRNAs mentioned above were obtained by the miRWalk. Furthermore, 497 overlapped genes were identified by intersecting the miRNA targeted genes and DEGs from TCGA, as Fig. 5b showing.

### Function enrichment analyses

**Figure 6:**
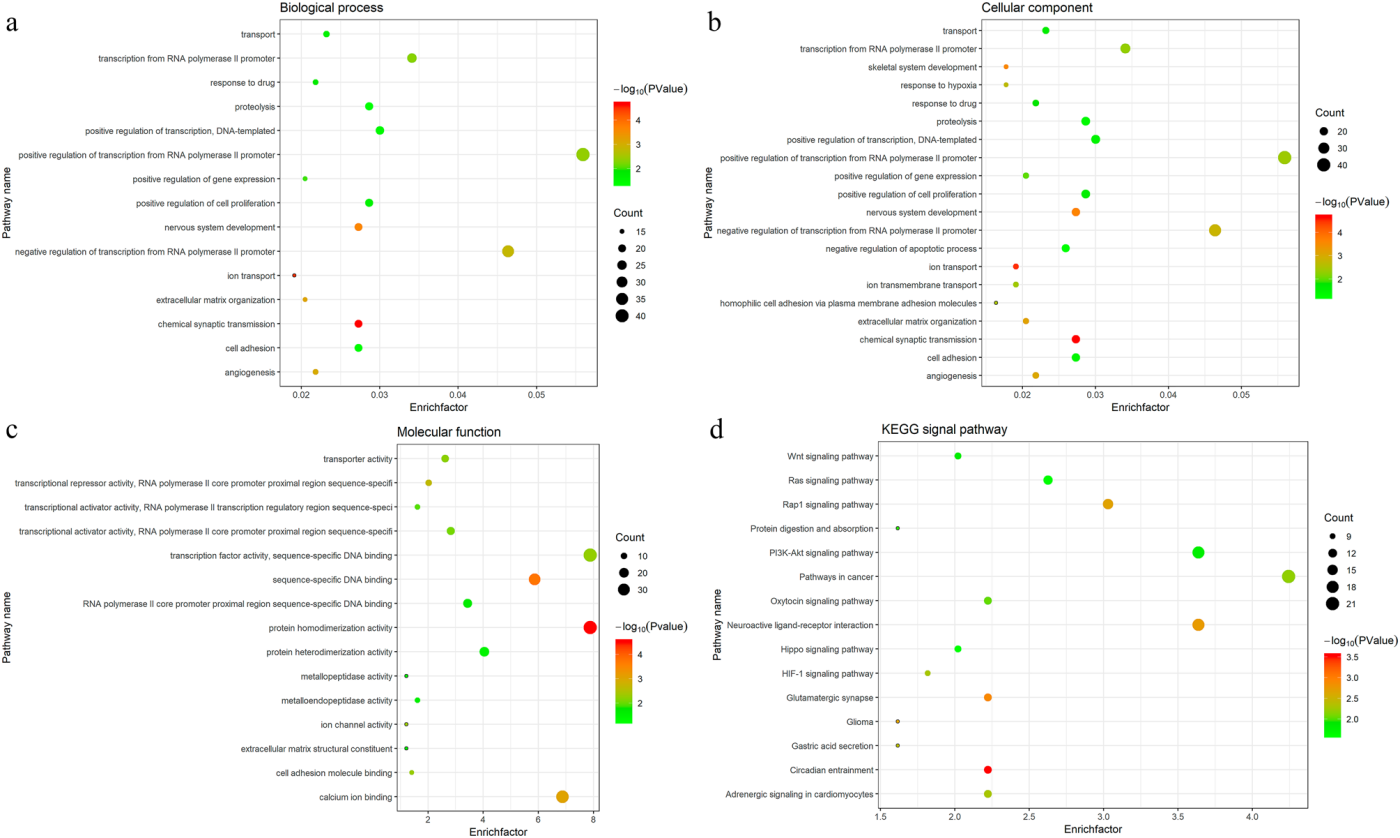
Dot plot of function enrichment analysis. (**a**) Biological process analysis. (**b**) Cellular component analysis. (**c**) Molecular function analysis. (**d**) KEGG pathway analysis. The color intensity of the nodes shows the degree of enrichment of this analysis. The enrich-factor is defined as the ratio of the differential genes in the entire genome. The dot size represents the count of genes in a pathway.

To understand the biological roles and potential functions of the 497 overlapped genes, GO analysis, which including biological process (BP), molecular function (MF), and cellular component (CC), and KEGG signal pathway enrichment analysis had been performed. GO analysis showed that in BP terms, these overlapped genes were mainly enriched in positive regulation of transcription from RNA polymerase II promoter, negative regulation of transcription from RNA polymerase II promoter, transcription from RNA polymerase II promoter, and positive regulation of transcription, DNA-templated. For MF, overlapped genes were mainly enriched in protein homodimerization activity, transcription factor activity, sequence-specific DNA binding, calcium ion binding, and sequence-specific DNA binding. For CC, these overlapped genes were mainly enriched in integral component of membrane, plasma membrane, integral component of plasma membrane, and extracellular region. The KEGG signal pathway enrichment analysis found that DEGs are mainly enriched in pathways such as Pathways in cancer, Neuroactive ligand-receptor interaction, PI3K-Akt signaling pathway, Rap1 signaling pathway, and Ras signaling pathway. The results are shown in Fig. 6.

### Identification of 10 hub-genes

**Figure 7:**
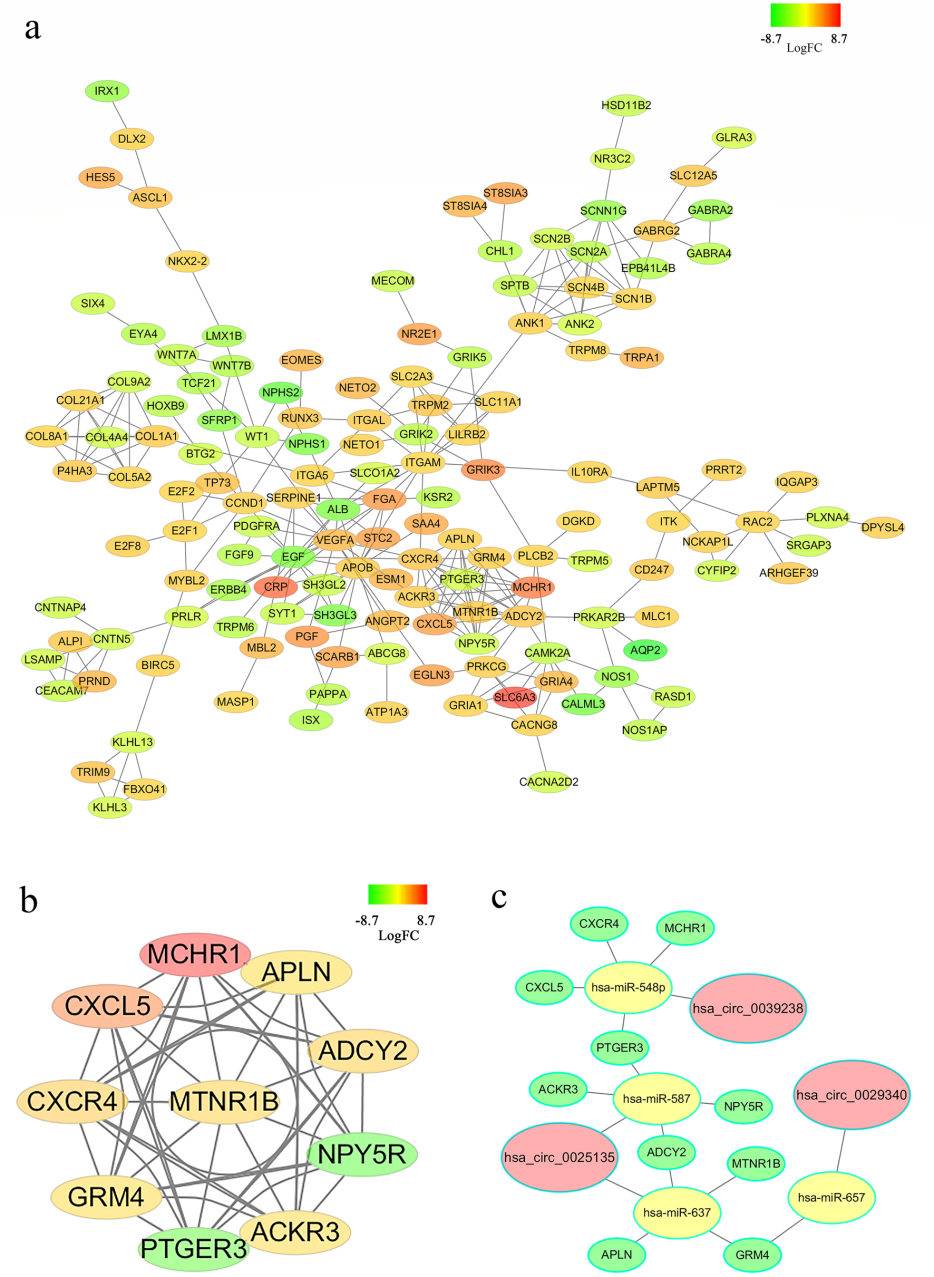
(**a**) A network of overlapped genes’ interaction. Gradual changes in color represent differences in the expression levels of different genes of ccRCC. (**b**) A network of 10 hub-genes. Gradual changes in color represent differences in the expression levels of different genes of ccRCC. (**c**) A network showing the interactions of circRNAs/miRNAs/hub-genes. circRNAs, miRNAs, hub-genes are shown in red, yellow, and green respectively).

The functions of these overlapped genes had been analyzed above. To further explore the effect of circRNA/miRNA regulatory networks on gene expression levels in ccRCC, a PPI network was constructed, and some hub-genes, which screened from this PPI network by applying some bioinformatics algorithms, were obtained. By utilizing STRING and Cytoscape, a PPI network consisting of 177 nodes and 330 edges to display the interactions among the 497 mRNAs (Fig. 7a). Then a subnetwork (MCODE score:9.0) with ten nodes and 45 edges was selected, which revealed the critical roles of the ten genes (PTGER3, ADCY2, APLN, CXCL5, GRM4, MCHR1, NPY5R, CXCR4, ACKR3, MTNR1B) in clear cell renal cell carcinoma (Fig. 7b). After this, a network about the association between these circRNA, miRNAs and hub-genes were built (Fig. 7c). 13 circRNA/miRNA/mRNA regulatory axis, including hsa_circ_0029340/has-miR-657/GRM4 axis, hsa_circ_0025135/has-miR-587/PTGER3 axis, hsa_circ_0025135/has-miR-587/ADCY2 axis, hsa_circ_0025135/has-miR-587/ACKR3 axis, hsa_circ_0025135/has-miR-587/NPY5R axis, hsa_circ_0025135/has-miR-637/APLN axis, hsa_circ_0025135/has-miR-637/GRM4 axis, hsa_circ_0025135/has-miR-637/MTNR1B, hsa_circ_0039238/has-miR-548p/PTGER3 axis, hsa_circ_0039238/has-miR-548p/HAPLN1 axis, hsa_circ_0039238/has-miR-548p/CXCL5 axis, hsa_circ_0039238/has-miR-548p/MCHR1 axis, hsa_circ_0039238/has-miR-548p/CXCR4 axis, extracted from this network.

### Effects of hub-genes on overall survival

**Figure 8:**
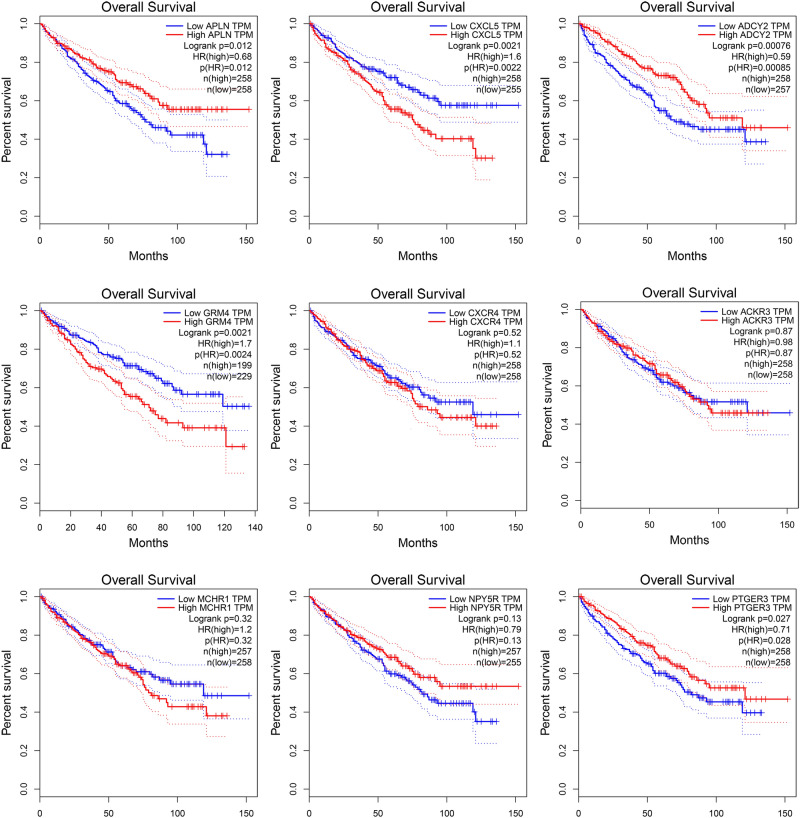
The overall survival time of 9 hub-genes.

The hub-genes and it’s predicted regulated pathways had been found out. And now the overall survival time of patient's with ccRCC will be displayed, which is the most concerning thing by all clinicians. The OS of these hub-genes are exhibited in Fig. 8, excluding MTNR1B which did not have sufficient data to go on survival analysis. Total five genes were found to have significant effects on overall survival. The overexpression of these genes, PTGER3, ADCY2, and APLN, can prolong the overall survival and may act as a protective factor, but the down-regulated genes, CXCL5 and GRM4, have adverse effects on OS, may act as risk factors.

### Construction of a circRNA–miRNA–mRNA network

**Figure 9:**
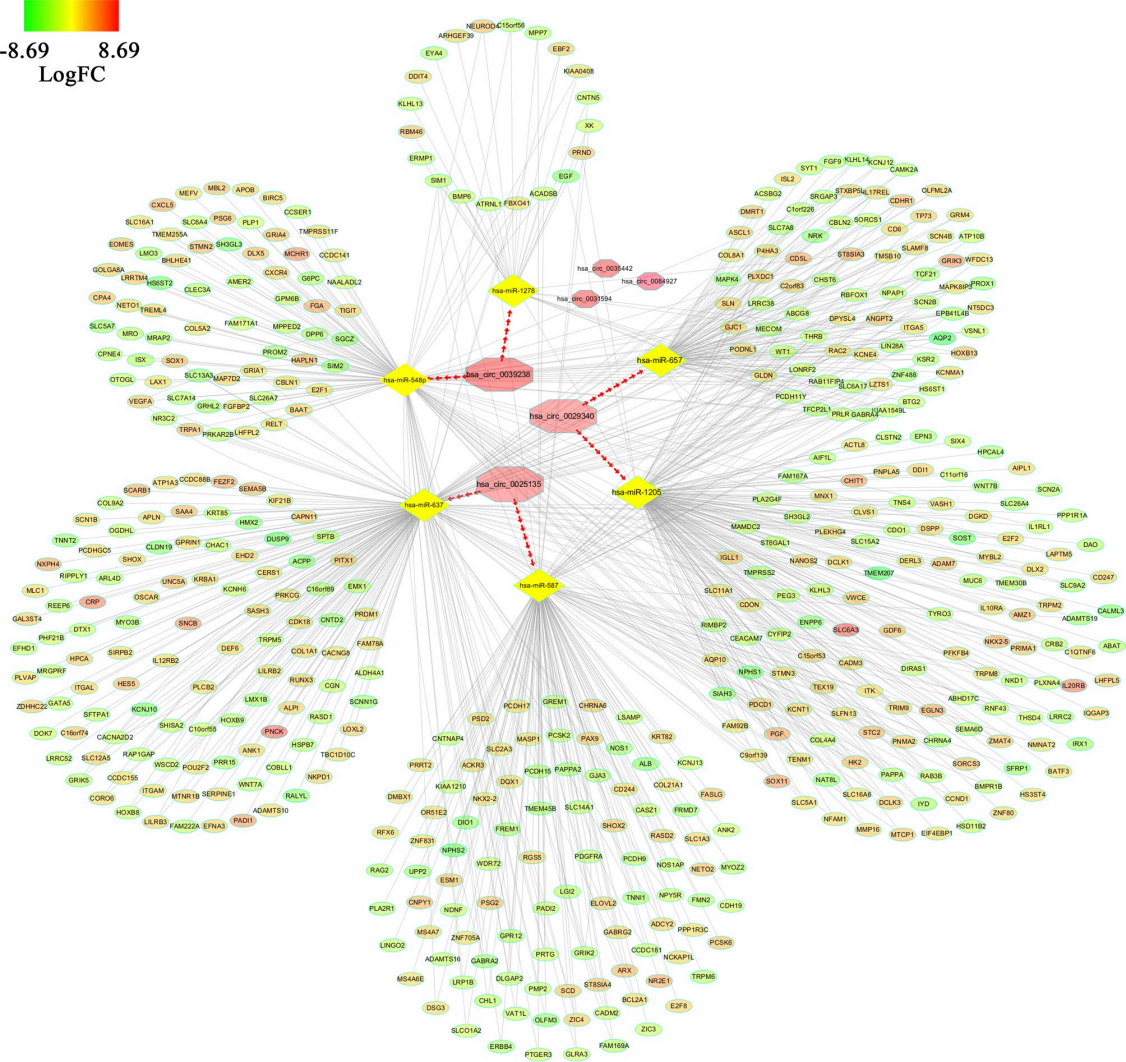
A network of circRNA/miRNA/mRNA. Octagon represents circRNA, Diamond represents miRNA, Oval represents mRNA. Gradual changes in color represent differences in the expression levels of different genes of ccRCC.

To present the relationship between circRNA, miRNA, and mRNA, a circRNA/miRNA/mRNA network was constructed through combining the circRNA/miRNA interactions and the miRNA/mRNA interactions by using Cytoscape, shown as Fig. 9. It provided an expression about the connections between the 3 DECs (hsa_circ_0029340, hsa_circ_0025135, hsa_circ_0039238), the 6 miRNAs (miR-1205, miR-657, miR-587,miR-637, miR-1278,miR-548p) and the 497 mRNAs.

### Candidate compounds from CMap

The regulated network of circRNAs, miRNAs, mRNAs, had been constructed, and now some candidates or drugs, which may have effects on ccRCC, will be forecasted. The candidate compounds were predicted by CMap, showing in Table 2. The enrichment correlation coefficient calculated by the correlation coefficient in all instances ranges from 1 to + 1. The score is positive, indicating that a small molecule compound or drug has a similar or co-directional relationship with a particular biological process; score is negative, indicating that a small molecule compound or drug has an opposite or antagonistic link to a particular organism. The *P* value was evaluated for the significance of the enrichment correlation coefficient and the smaller the value, the greater the credibility^25^.

**Table 2:** A potential compound identified by Cmap for ccRCC.

Cmap name	Enrichment score	Dose (μm)	Cell	Up score	Down score
josamycin	− 0.411	5	MCF7	− 0.618	− 0.114
josamycin	− 0.411	5	HL60	− 0.775	− 0.136
josamycin	− 0.411	5	PC3	− 0.661	− 0.124

## Pharmacogenomics analysis for hub genes to find potential drug

By exploring the website PharmGkb, the above ten hub-genes were selected for pharmacogenomic analysis to find some potential drugs and complement the results of CMap. The results are showing in Table 3. Bevacizumab can treat Colorectal Neoplasms efficiency, which may be related to the expression of CXCR4. And capecitabine can also work with colorectal cancer reduced progression-free survival but slightly. These two drugs mentioned above are all associated with cancer, but hmg coa reductase inhibitors and Ace Inhibitors, Plain are not associated with cancer as the annotation from the website introduces.

**Table 3:** Four potential drugs identified by PharmGKB for ccRCC.

Genes	SNP	Drug	Significant	*P* value	Association	References
ADCY2	rs4702484	Capecitabine	Yes	0.018	In patients receiving capecitabine monotherapy CC carriers showed slightly reduced progression-free survival (CC 6.2 vs. CT 8.0 months; *P* = 0.018)	PMID: 25815774
ADCY2	rs4702484	Capecitabine	No	0.229	Analyzing the entire cohort of capecitabine monotherapy (N = 126) and Combination therapy (N = 139) no association for genetic markers with progression-free survival was found	PMID: 25815774
CXCL5	rs352046	Hmg coa reductase inhibitors	Yes	0.0009	Genotype CC is associated with increased response to hmg coa reductase inhibitors in people with Acute coronary syndrome as compared to genotypes CG + GG	PMID: 18769620
PTGER3	rs11209716	Ace Inhibitors, Plain	Yes	0.002	Allele C is associated with decreased risk of Cough when treated with Ace Inhibitors, Plain in people with Hypertension as compared to allele T	PMID: 17496729
CXCR4	rs2228014	Bevacizumab	Yes	0.029	Genotypes AA + AG is associated with decreased progression-free survival when treated with bevacizumab in people with Colorectal Neoplasms as compared to genotype GG	PMID: 27503580

## Discussion

An increasing number of researches have demonstrated that circRNAs and miRNAs play important roles in cancer biological recently. Experiments had demonstrated that miRNAs are closely related to the proliferation, migration, and invasion, and circRNAs can regulate these processes via miRNAs in ccRCC^26–28^. Some studies also demonstrated that circRNA/miRNA has a strong association with diseases, especially with cancer, by utilizing advanced computational methods such as KATZ algorithm, Locality-Constrained Linear Coding algorithm, inductive matrix completion, decision tree^29–35^ . Therefore, circRNAs and miRNAs can apply as a therapeutic method or a biomarker of diagnosis. In this study, 6 DECs (hsa_circ_0029340, hsa_circ_0039238, hsa_circ_0031594, hsa_circ_0084927, hsa_circ_0035442, hsa_circ_0025135) were selected at the first step. To our knowledge, hsa_circ_0084927 had been demonstrated that it is involved in the development of lung adenocarcinoma-associated malignant pleural effusion, but the other 5 circRNAs had not been studied yet^36^. These five circular RNAs were first discovered that are abnormally expressed in ccRCC, and they have the potential to be excellent biomarkers or potential therapeutic targets. As a kind of highly conserved endogenous RNA (ceRNA), circRNA has been confirmed to have a function as "sponge" to absorb corresponding miRNA by interacting with miRNA binding sites and, thus, playing a role in regulating genes expression indirectly. Among the 6 DECs above, 3 circRNAs (hsa_circ_0029340, hsa_circ_0025135, hsa_circ_0039238) were ascertained as ceRNA to regulate the expression of 6 miRNAs (miR-1205, miR-657, miR-587, miR-637, miR-1278, miR-548p). However, based on the ceRNA theory, no miRNAs related to the other three circRNAs were found, which may play other roles, such as coding protein, interaction with RNA binding protein, or modulating the stability of mRNAs so on. Among the 6 miRNAs identified, miR-657, miR-587 was confirmed to act as tumor-promoting molecules in some cancer types^37–41^. Some research have found that miR-657 overexpressed in lung cancer, cervical squamous cell carcinoma, larynx carcinoma, hepatocellular carcinoma, and it can promote carcinoma cells abilities of tumorigenesis, proliferation and invasion by some complex targeting pathways^37,40^. And For miR-587, it can antagonize 5-FU-induced apoptosis and confers drug resistance by regulating PPP2R1B expression in colorectal cancer, and it is related to the survival time of glioblastoma multiforme patients^41,42^. In contrast, mir-1205, miR-637, and miR-548p act as tumor suppressor molecules. MiR-1205 can target some downstream gene sits to inhibited and promote cell proliferation and invasion in some cancers ^43–45^. For miR-637, many studies suggest it act as a protective factor to suppress the cancer cells proliferation, invasion and migration by targeting on regulating the expression of AKt1, RING1 or NUPRI in hepatocellular carcinoma, colorectal cancer, glioma, and cervical cancer^45–49^. MiR-548p decreases Hepatic Apolipoprotein B Secretion and Lipid Synthesis, acting at HBx/HNF4A/miR-548p/HBXIP pathway that controls hepatoma cell growth and tumorigenesis of hepatocellular carcinoma^50^. But there are no studies having found that miR-1278 plays an important regulatory role in tumors. In general, the findings of this study about these miRNAs are similar to other studies, and these miRNAs may play an important role in the development of ccRCC.

CircRNAs affect the expression of genes by acting at miRNA, as shown above. To further explore the effects of circRNA on gene expression by acting at miRNA, 497 overlapping genes were collected to go for function enrichment analyses. These genes were enriched mainly in the biological process about the regulation about transcription and cell proliferation. Meanwhile, KEGG analysis showing that these genes were mainly enriched in some cancer-related pathways such as Pathways in cancer, PI3K-Akt signaling pathway, Rap1 signaling pathway, and Ras signaling pathway. Furthermore, these pathways have been shown to have an interaction in some cancers, which may indicate that these circRNAs explored in this study may exert the same or related regulatory functions by acting on the circRNA/miRNA/mRNA axis. PI3K/Akt signaling pathway is an important pathway to regulate cancer proliferation, adhesion, migration, invasion and angiogenesis. It acts at downstream targets, as Forkhead box O transcription factors (FoxO), mammalian target of rapamycin (mTOR), to stimulate expression of death receptor ligands and enhance Vascular endothelial growth factor (VEGF) secretion^51,52^. Meanwhile, PI3K/Akt signaling pathway can be activated by RAS or Rap1 pathway, and it can interact with gene P53 to promote the function of enabling gene repair and maintaining gene stability^53^. Moreover, Rap1 is activated by upstream signaling molecules (as calmodulin, cAMP) and tyrosine kinase (as PKA PKC), acting on downstream molecular markers (such as B-raf PAPL) to regulate gene expression, cell proliferation, adhesion, etc^54,55^. In summary, the function and signaling pathways of these genes are related to the occurrence and development of tumors, which have been confirmed in other studies. Therefore, these genes, which are regulated by circRNAs indirectly, play an essential role in the signal pathway of ccRCC.

In this study, hub-genes (PTGER3, ADCY2, APLN, CXCL5, GRM4, MCHR1, NPY5R, CXCR4, ACKR3, MTNR1B) were obtained, and some circRNA/miRNA/mRNA regulatory axis about these hub-genes were constructed which may help researchers build a more systematic, more profound about the regulatory network. Among these hub-genes, PTGER3, CDCT2, APLN, CXCL5 and GRM4 show significant effects on overall survival time between ccRCC tissue group and normal tissue group. PTGER3, CDCT2 and APLN have positive effects on overall survival time, but CXCL5 and GRM4 have negative effects on it. Research demonstrated that APLN could interact with APLN receptor, which is a G-protein-coupled receptor, which may influence the aggressive of ccRCC and the effect of immune therapy^56^. And C-X-C chemokine receptor 4 (CXCR4) is the major chemokine receptor in solid tumors. Increased CXCR4 expression was associated with more aggressive tumor behavior in RCC patients, especially in ccRCC subtypes, due to their more metastatic behavior^57^. However, to our best knowledge, the rest hub-genes in ccRCC had not been investigated.

Clear cell renal cell carcinoma (ccRCC) is one of the most drug-resistant malignancies. Exploring potential compounds or drugs that may have a therapeutic effect on ccRCC is necessary. Cmap and Pharmacogenomics analysis had been exploited to find potential compounds and drugs. Josamycin is a naturally produced antibiotics that have a 16-membered macrocyclic lactone ring predicted by Cmap. A study had demonstrated that Josamycin can suppress the development of altered liver cell foci but not indicted this compound has anti-cancer function^58^. Meanwhile, a study demonstrated that 14-membered ring macrolide antibiotics, roxithromycin and clarithromycin, have a significant inhibitory effect to mouse B16 melanoma cell on tumor angiogenesis, tumor growth, and metastasis. However, t a 16-membered ring macrolide, as josamycin, did not show any inhibitory effect on these ways when at the same dose^59^. So, more studies are needed to illustrate whether josamycin can act as anti-tumor function and its mechanism of action. Capecitabine is targeting to the SNPs of rs4702484 of ADCY2, and reduced progression-free survival but slightly. However, the effect of chemotherapy for clear cell renal cancer is poor. Capecitabine is seldom used in treating renal cancer, sometimes used in combination with targeted drug therapy^60–62^. Bevacizumab targeting to the SNPs of rs228014 of CXCR4, exerting anti-tumor effect. Bevacizumab is a monoclonal antibody that inhibits vascular endothelial growth factor and is used to treat various metastatic cancers. It has the ability to bind to the VEGF receptor (VEGFR), which are on the surface of endothelial cells and are membrane-bound tyrosine kinase receptors responsible for specific downstream survival and proliferation pathways^63^. Also, bevacizumab is commonly used in treating metastatic ccRCC, combining it with other drugs, as interferon, erlotinib^64–67^. Hmg-coa reductase inhibitors have been confirmed to have anti-cancer effects in some cancers, but whether it has the same effects on ccRCC had not been confirmed^68^. It may be a potential therapeutic drug and need researches to test and verify. However, for Angiotensin Converting Enzyme (ACE)-Inhibitory, whether it has an anti-tumor effect is still unclear, some recent studies had demonstrated that Angiotensin Converting Enzyme (ACE) Inhibitory could induce apoptosis of cancer cell^69,70^. But some studies also demonstrated that it might increase the risk of canceryy^71^. To sum up, these compounds and drugs, which were predicted by this study, maybe the right choice for the treatment of ccRCC.

Several limitations should be considered. The construction of circRNA/miRNA/mRNA regulatory networks and the prediction of therapeutic drugs were all relying on a series of bioinformatics algorithms and databases. A large number of experiments are needed to verify the accuracy of these prediction conclusions. In addition, in the choice of DECs, the selection criteria with higher credibility are adopted. Although the specificity of the prediction results is improved, the sensitivity is insufficient in this study. In this paper, the latest and most reasonable algorithms were selected, but random errors and selection bias cannot be avoided. In future research, we will verify our predictions in cell lines and in entity samples.

## Conclusion

In this study, we firstly constructed a circRNA/miRNA/mRNA regulatory network based on the theory of ceRNA through using some bioinformatic tools and genomics databases. Furthermore, the functions of these circRNA/miRNA/mRNA links are also had been predicted. In addition, some compounds and drugs for ccRCC are predicted which may be the potential treatment candidates. All this work can improve our understanding of potential pathogenesis in ccRCC, and it has directive functions for further research works. However, all these results were speculated by various bioinformatics analyses, and it still needs to be further confirmed .

## Data Availability

The authors declare that the data supporting the findings of this study are available within the article.
